# Quantitative liver surface nodularity score based on imaging for assessment of early cirrhosis in patients with chronic liver disease: A protocol for systematic review and meta-analysis

**DOI:** 10.1097/MD.0000000000023636

**Published:** 2021-01-29

**Authors:** Yuhao He, Yujia Yan, Sunfu Zhang

**Affiliations:** a Department of Neurosurgery, Chengdu Third People's Hospital, Chengdu, Sichuan Province, 610031; b Department of Neurosurgery, Tianjin Huanhu Hospital, Tianjin, 300000, China.

**Keywords:** cirrhosis, liver surface nodularity, meta-analysis, protocol, quantitative, systematic review

## Abstract

**Background:**

Early stage of cirrhosis is of great value in the diagnosis and management in patients with chronic liver disease (CLD). Recent studies have shown that quantitative liver surface nodularity (LSN) score based on imaging techniques can be used to predict the early cirrhosis stage noninvasively, with varied diagnostic accuracy and limited sample size. Hence, this study will evaluate the diagnostic accuracy of LSN in the prediction of early cirrhosis.

**Methods:**

We will conduct a comprehensive search in PubMed, Web of Science, Cochrane Library, and Chinese biomedical databases to identify eligible studies. The literature screening, data extraction, data analysis, and quality assessment will then be carried out. The summary receiver-operating-characteristic (ROC) and pooled sensitivity, specificity will be calculated to summarize the diagnostic performance of LSN using a random-effect model. A meta-regression analysis will be performed to investigate the underlying cause of the heterogeneity.

**Results:**

This study will evaluate the diagnostic accuracy of LSN score in the identification of early cirrhosis, which may further determine whether this method can be used as an alternative in the assessment of CLD patients.

**Conclusions:**

This study will help to determine the diagnostic accuracy and summarize the recent evidence on this issue.

**Study registration:**

INPLASY2020100096.

## 1 Introduction

Chronic liver disease (CLD) is the major cause of global healthy problem contributing to high morbidity and mortality, patient at the end stage of CLD can be associated with a series of devastating complications.^[1]^ Nevertheless, early stage of cirrhosis or fibrosis might be reversible and is of great value in diagnosis and management of CLD patients,^[2]^ according to the Brunt & Kleiner, Ludwig, METAVIR, SAF or Scheuer scoring system,^[3,4]^ the early cirrhosis stage is defined as F4.

Liver biopsy is currently considered as the reference standard for liver cirrhosis and staging of fibrosis, however, the invasive nature, high cost-effectiveness, sampling errors, variability in assignment of pathologic stage may limit its wide utilization.^[5]^ Thus, noninvasive methods for assessing cirrhosis and staging fibrosis have been developed and validated in the recent years, such as the noninvasive serum-based markers, elastography imaging with ultrasound (US), or magnetic resonance imaging (MRI) techniques, these techniques cannot replace histologic assessment due to the insufficiently accuracy or dedicated equipment requirement.^[6–8]^

There is growing evidence that the morphologic change of liver surface nodularity (LSN) based on imaging modalities is associated with the severity of CLD, which has been applied in US,^[9–11]^ computed tomography (CT), and MRI,^[12,13]^ recent studies demonstrated that quantitative LSN score using these novel imaging techniques has the potential to predict the early cirrhosis stage (F4).^[14,15]^ Whereas, the results of these studies are insufficiently robust with a small sample size, and no clear consensus has been reached about the diagnostic accuracy of LSN score with varied cut-off values; in addition, there has been no previous published meta-analysis evaluating the diagnostic performance of quantitative LSN score based on imaging procedures. Hence, in this study, we will perform a systematic review and meta-analysis of the diagnostic performance of LSN score for predicting early liver cirrhosis and to further determine the cut-off value in clinical practice.

## 2 Materials and methods

### 2.1 Study registration

This protocol report will be structured according to the Preferred Reporting Items for Systematic Reviews and Meta-analysis Protocols (PRISMA-P) statement.^[16]^ It has been registered on the INPLASY2020100096.

### 2.2 Eligibility criteria

#### 2.2.1 Type of study

High quality clinical cohort or case–control studies will be included in this study.

#### 2.2.2 Type of patients

The patients should be those who had undergone CT for measurement of LSN and diagnosed as early cirrhosis.

#### 2.2.3 Intervention and comparison

This study will compare LSN score with pathology of liver biopsy for diagnosing early cirrhosis.

#### 2.2.4 Type of outcomes

The primary outcomes include sensitivity, specificity, diagnostic odds ratio, and the area under the curve of the summary receiver operating characteristic and the optimal cut-off value.

### 2.3 Search methods

Relevant studies in PubMed, EMBASE, Cochrane Library databases, and Web of Science will be systematically searched from database inception to 14 October 2020, using the search terms as following:((((cirrhotic[Title/Abstract]) OR (cirrhosis[Title/Abstract])) OR (fibrosis[Title/Abstract])) OR (((((hepatic venous pressure gradient[Title/Abstract]) OR (portal hypertension[Title/Abstract])) OR (HVPG[Title/Abstract])) OR (clinically significant portal hypertension[Title/Abstract])) OR (CSPH[Title/Abstract]))) AND (((liver surface nodularity[Title/Abstract]) OR (liver surface nodule[Title/Abstract])) OR (hepatic surface nodularity[Title/Abstract])). No filters will be applied (i.e., language), all eligible literatures will be retrieved and their reference of the initial studies will be checked for additional relevant publications.

### 2.4 Inclusion criteria

We will export all trials into Endnote X9 according to the inclusion criteria listed as follows:

(1) patients with CLD diagnosed with cirrhosis,
(2) liver biopsy is considered as the reference standard,
(3) liver surface nodularity measurement is conducted to evaluate liver cirrhosis,
(4) studies have enough data to obtain a diagnostic 2 × 2 table of test performance (true-positive (TP), false-positive (FP), true-negative (TN), and false-negative (FN) diagnostic results).

Two reviewers will screen the titles and abstracts of literature independently to identify potential eligibility, then, the full texts of potentially eligible studies will be reviewed for final inclusion.

### 2.5 Data extraction

The following information will be extracted from each included study: first author's last name, publication year, country, study design, mean age, male/female ratio, aetiology of CLD, reference standard and histopathological hepatic fibrosis staging system, imaging modalities, time interval between reference and LSN measurement, LSN measurement software, sample size, TP, FP, FN, TN, area under the receiver operating characteristic curve (AUROC), and cut-off values. TP, FP, FN, TN will be extracted or calculated according to reported sensitivity and specificity to form a diagnostic 2 × 2 table.

### 2.6 Quality assessment

Two independent reviewers will conduct the literature search, study selection, data extraction, and quality assessment, with a third reviewer adjudicating on disagreements. The quality assessment will be evaluated with the revised Quality Assessment of Diagnostic Accuracy Studies-2 (QUADAS-2) tool.^[17]^ Each individual term is categorized as “yes” if it was reported, “no” if not reported, or “unclear” if information was not enough to reach a conclusion. Quality assessment will be performed with Review Manager 5.3.

### 2.7 Statistical analysis

Stata V.15.1 (StataCorp LP, College Station, TX) will be used for statistical analysis, the summary sensitivity, specificity, and the corresponding 95% CIs will be calculated using data for the numbers of TP, FP, FN, TN and a coupled forest plot will be constructed, thereafter, a summary receiver operating characteristic (SROC) curve with a 95% CI and summary AUROC can be obtained. The threshold effect will be evaluated using the Spearman correlation coefficient, a threshold effect might exist with *P* ≤ .05. Heterogeneity among included studies will be assessed using the Cochran's *Q* test (*P*) and inconsistency index (*I*^2^), a *P* < .1 or *I*^2^ > 50% indicated significant heterogeneity. To investigate the underlying cause of the heterogeneity, a meta-regression analysis will be conducted by investigator.

### 2.8 Additional analysis

#### 2.8.1 Subgroup analysis and sensitivity analysis

We will further conduct a subgroup analysis and a sensitivity analysis to investigate potential sources of heterogeneity by comparing different covariates or excluding the low-quality studies.

#### 2.8.2 Reporting bias

We will conduct funnel plots and associated regression tests to evaluate the publication bias if necessary.

#### 2.8.3 Ethics and dissemination

This study does not need ethical approval because it will not be performed on individual patient directly, we hope to publish our study on a peer-reviewed journal.

## 3 Discussion

Chronic liver disease and the subsequent liver cirrhosis are considered as a major cause of global healthy problem and is generally irreversible in the end stage.^[1]^ Current modalities including elastography, laboratory biomarkers, and subjective LSN stage system are not qualified to replace the reference standard of invasive liver biopsy, as a result of limited accuracy, dedicated equipment requirement, and interobserver variability.^[18]^ Recent studies have developed and validated a new semiautomated software equipped with quantitative tool for deriving objective scores of LSN on multidetector computed tomography (MDCT) and MRI for predicting early cirrhosis,^[12,13,19]^ obviating the invasive liver biopsy and the drawbacks of previous methods with varied diagnostic performance. To our knowledge, there has been no previous meta-analysis evaluating the diagnostic performance of quantitative LSN score for predicting early cirrhosis based on imaging procedures, additionally, no consensus has been reached about the diagnostic accuracy of LSN with varied cut-off values. In the study, we will perform a systematic review and meta-analysis of the diagnostic performance of LSN score for predicting early liver cirrhosis and to further determine the cut-off value in CLD patients, and we hope to find more robust evidence of noninvasive assessment of the early cirrhotic stage that may assist clinical decision making process.

## Author contributions

**Conceptualization:** Yuhao He.

**Data curation:** Yuhao He.

**Investigation:** Yuhao He.

**Methodology:** Yuhao He, Yujia Yan.

**Software:** Yujia Yan.

**Supervision:** Sunfu Zhang.

**Writing – original draft:** Yuhao He.

**Writing – review & editing:** Yuhao He, Yujia Yan.

## Abbreviations

Abbreviations: AUROC = area under the receiver operating characteristic curve, CLD = chronic liver disease, CT = computed tomography, FN = false-negative, FP = false-positive, LSN = liver surface nodularity, MDCT = multidetector computed tomography, MRI = magnetic resonance imaging, PRISMA-P = Preferred Reporting Items for Systematic Reviews and Meta-analysis Protocols, QUADAS-2 = Assessment of Diagnostic Accuracy Studies-2, ROC = receiver-operating-characteristic, SROC = summary receiver operating characteristic, TN = true-negative, TP = true-positive, US = ultrasound.
